# Prevalence, Risk Factors, and Causes of Visual Impairment in an Elderly Chinese Uygur Population in Southern Xinjiang

**DOI:** 10.1155/2021/8843032

**Published:** 2021-04-07

**Authors:** Yi Sun, Guangming Jin, Mengting Yang, Jing Fu, Xueyi Chen, Bingsheng Lou

**Affiliations:** ^1^State Key Laboratory of Ophthalmology, Zhongshan Ophthalmic Center, Sun Yat-sen University, Guangzhou 510060, China; ^2^Department of Ophthalmology, The First Affiliated Hospital of Xinjiang Medical University, Urumqi 830054, China

## Abstract

**Purpose:**

To investigate the prevalence, risk factors, and major causes of visual impairment (VI) in an elderly Chinese Uygur population in southern Xinjiang.

**Methods:**

This was a population-based cross-sectional study. Participants aged 50 years and older from Haohan Country, Xinjiang Uygur Autonomous Region, were enrolled from August 2018 to December 2018 using cluster sampling. Participants underwent examinations including presenting visual acuity (PVA), pinhole vision, slit-lamp, intraocular pressure, and direct ophthalmoscopy. Participants' education and demographic information was collected by a questionnaire. The prevalence, risk factors, and major causes of vision loss were evaluated.

**Results:**

A total of 1465 participants (85.4% response rate) were enrolled. The mean age of the subjects was 59.1 ± 9.7 years. The prevalence of mild VI, moderate VI, severe VI, and blindness in the better eye was 13.3%, 12.8%, 2.9%, and 3.4%, respectively. The prevalence of low vision and blindness in this study was higher than that in Altay & Tacheng and Changji in northern Xinjiang, lower than that in Luxi, and similar to that in Tibet. The multiple logistic regression analysis showed that age, education level, and body mass index (BMI) were significantly associated with low vision and blindness (*P* ≤ 0.001, <0.05, 0.002, respectively). The major causes of low vision were cataract (42.6%), refractive error (19.6%), and glaucoma (12.6%), whereas the primary causes of blindness were cataract (34%), glaucoma (34%), and retinitis pigmentosa (10%).

**Conclusions:**

VI is an important public health issue among elderly Uygur individuals in the area, especially for those with low education levels. Cataract is the leading cause of low vision and blindness.

## 1. Introduction

The prevalence of visual impairment (VI) varies among different countries. For example, the prevalence of low vision and blindness is 10.3% and 1.66% in China [1], 21.7% and 3.8% in Ghana [2], and 0.37% and 0.05% in Germany, respectively [3]. Overall, people in low socioeconomic areas have an increased rate of VI [4] due to the limited affordability and accessibility of eye care services. In 1999, the global initiative “Vision 2020: The Right to Sight” was launched by the World Health Organization (WHO) and the International Agency for the Prevention of Blindness to eliminate avoidable blindness, and by 2012, blindness was prevented in an estimated 100 million people [5].

China is the most populous country in the world, with a population of more than 1.4 billion in 2019; approximately 39.4% of residents (552 million) live in rural areas. China is home to a multi-ethnic population, which includes Han and 55 other ethnic minority groups. Most studies on the prevalence of VI in China have been based on the Han population [6–9] except for a few studies on other minority populations, such as the Bai, Dai, and Zang populations [10–12]. Uygur is one of the major Chinese ethnic minority groups, with a population of approximately 10 million (2010), and 99% of them inhabit the Xinjiang Autonomous Region in northwest China. There were 2 studies conducted before 2015 on the different prevalence of VI in Xinjiang, namely, the China Nine-Province Survey in Changji demonstrating moderate VI in 7.41% of participants, severe VI in 0.61% of participants, and blindness in 1.33% of participants [13], and the other study in Altay and Tacheng demonstrating a prevalence of 3.6%, 1.2%, and 1.7%, respectively [14]. Both study sites are situated in northern Xinjiang, and the participants were not all Uygur. Haohan Country is located in the southern part of Xinjiang and has a different lifestyle, environment, and economic levels than those of northern Xinjiang. Moreover, the southern Xinjiang region is home to the largest Uygur population in China, and nearly all rural residents in this area are of Uygur ethnicity. To date, whether the prevalence of VI is different between these two areas in Xinjiang remains unknown.

The current study aims to present data on the prevalence of VI among an adult Chinese Uygur population in the southern Xinjiang Autonomous Region and to determine the associated risk factors and major causes of VI in this population. The information provided in this study may contribute to the accurate estimation of the disease burden and guide health policy planning and service delivery.

## 2. Methods

### 2.1. Study Population

Xinjiang is a multi-ethnic municipality in western China; the most prominent ethnicity is Uygur. We selected Haohan Country (a suburb of Kashi City) for this survey because its socioeconomic profile represents the Xinjiang Uygur minority groups. Based on information from the Residence Administrative Committee, the sampling frame was created using geographically defined clusters of about 1000 people (population older than 50 years accounts for about 20%). Villages with more than 1500 people were subdivided, and those with less than 500 people were combined. Each geographically defined cluster was regarded as a basic sample unit, and 49 basic sampling units were constructed using this method. Nine clusters from which about 1800 eligible subjects could be recruited were randomly selected. Eligible individuals were those aged ≥50 years who had been living in the selected areas for ≥6 months. People who had been away from the areas for more than 6 months and those who could not undergo the examinations were excluded from the study. The study was approved by the ethics committees of the Zhongshan Ophthalmic Center (no. 2020KYPJ075) and the First Affiliated Hospital of Xinjiang Medical University (no. 20170214–101) and adhered to the tenets of the Declaration of Helsinki.

The sample size was calculated according to the following formula: *N*=(*Z*)^2^(*P*)(1 − *P*)/[(*B*)(*P*)]^2^, in which P is the estimated prevalence of low vision and blindness (based on the results of the China Nine-Province Survey). B is an error bound with 95% confidence (20% was used in this study). The examination response rate was assumed to be 80% with a design effect of 1.5 to account for inefficiencies associated with the cluster sampling design.

Listings of households within each cluster with the names of residents aged 50 years or older were obtained from village registers, followed by door-to-door visits carried out by enumeration teams. Residents were enumerated by name, age, sex, education level, and spectacle usage. Residents temporarily absent during the household visit were included in the enumeration. Unregistered people aged 50 years or older were enumerated and included in the study sample if they had been residing in the household for more than 6 months.

### 2.2. Examinations

The examinations were conducted from August 2018 to December 2018. Door-to-door visits were performed, and a standard questionnaire was used to collect the following data from participants: general information (age, sex, nationality, education level, smoking status, drinking status, systemic disease history, ophthalmic disorder history, and surgery history) and highest level of education were obtained (uneducated, ≤primary school, junior middle school, and high school or higher). Systemic examinations included body height and weight, from which body mass index (BMI) was calculated.

An Early Treatment Diabetic Retinopathy Study (ETDRS) chart (Precision Vision, Inc., La Salle, IL, USA) was used to evaluate visual acuity (VA). The presenting VA (PVA) of each eye, which was determined based on the participants' available correction, was recorded, and followed by pinhole vision examination. The PVA of 100 participants was tested by two ophthalmologists and compared to assess their consistency, and the kappa was 0.9. Any discrepancy was discussed until a consensus was reached. Advice from a third ophthalmologist (BSL) was sought if necessary. Other examinations included slit-lamp biomicroscopy (SL-1E, Topcon), intraocular pressure (IOP) measurement using noncontact tonometry (CT-60, Topcon), and direct ophthalmoscopy (11710, Welch Allyn, USA). Participants with suspected glaucoma were referred to the First Affiliated Hospital of Xinjiang Medical University to receive the visual field test (Humphrey Field Analyzer II 750; Carl Zeiss Meditec, Dublin, CA).

### 2.3. Definitions of Visual Impairment

The WHO's definition of VI was used to classify patients [13]. Blindness was defined as a VA worse than 20/400 in the better eye. Severe VI was defined as a VA worse than 20/200 but better than 20/400 in the better eye. Moderate VI was defined as a VA worse than 20/63 but better than 20/200 in the better eye. Mild VI was defined as a VA worse than 20/40 but better than 20/63 in the better eye, and normal vision was defined as a VA better than 20/40 in the better eye. Low vision was defined as a VA worse than 20/63 but better than 20/400 in the better-seeing eye. The prevalence of VI was estimated based on the PVA in the better eye.

### 2.4. Major Causes of Visual Impairment

Participants with a PVA worse than 20/63 in the better eye were assigned a major cause of VI. For eyes with 2 or more reasons for VI, the disease with the greatest clinically significant impact on VA was considered the major cause. The ophthalmologist team discussed any disagreement until a consensus was reached.

The Lens Opacities Classification System III was used to diagnose cataract, which was considered the principal cause of VI when the cataract was commensurate with VI.

Age-related macular degeneration (AMD) was defined according to the Wisconsin Age-Related Maculopathy Grading System [15]. Refractive error was diagnosed if the pinhole vision was improved. Highly myopic retinopathy was defined as in subjects with scleral staphyloma and typical degenerative myopic fundus changes. Besides, the diagnoses of retinal venous occlusion, retinitis pigmentosa, pterygium, optic atrophy, and others diseases followed the clinical standards.

### 2.5. Statistical Analysis

Statistical analyses were performed with SPSS 23.0 advanced statistical software (SPSS Inc. Chicago, IL). The overall prevalence of VI was evaluated. The Kolmogorov–Smirnov test was used to check normality. The demographic variables were analyzed using Analysis of Variance (ANOVA) or the Kruskal–Wallis test. The categorical variables were analyzed using Chi-Squared test. Multivariate-adjusted logistic regression analysis of risk factors for the low vision and blindness was also performed. The odds ratios (ORs), 95% confidence intervals (CIs), and *P* values in the univariate and multiple logistic regression analyses were calculated. Differences were considered statistically significant when the *P* value was less than 0.05.

## 3. Results

**Figure 1:**
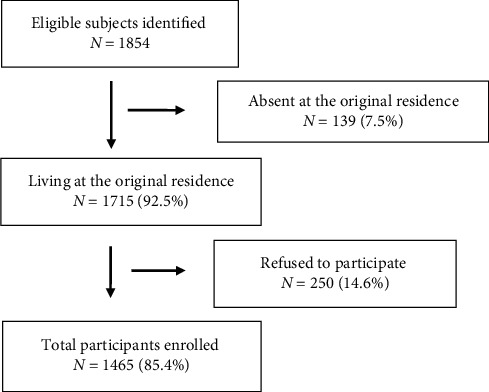
Workflow of participants' inclusion and exclusion.

The flowchart of participant inclusion and exclusion is shown in Figure 1. There were 1854 subjects aged 50 years or older at the time of the investigation. Among these, 1715 were eligible (based on village registers, 139 had moved away from their original residence). Finally, a total of 1465 subjects (85.4% response rate) agreed to participate in the study. The mean age of the participants was 59.1 ± 9.7 years. Among them, 673 (46%) were male and 792 (54%) were female.

The demographic characteristics of the participants with different VIs are shown in Table 1. There was a significant difference among the types of VI according to age (*P* ≤ 0.001), education level (*P* ≤ 0.001), and BMI (*P* ≤ 0.001), which indicated elder people with less education and lower BMI would be vulnerable to VI.  Table 2 shows that the prevalence of normal vision, mild VI, moderate VI, severe VI, and blindness was 67.6%, 13.3%, 12.8%, 2.9%, and 3.4%, respectively.

Comparisons between the prevalence of VI in this study and other minority population-based studies are listed in Table 3. In this study, the prevalence of low vision was higher than that in the Lhasa, Altay and Tacheng, and Changji eye studies [11, 13, 14]. Moreover, it was similar to that in the Luxi eye study [13]. The prevalence of blindness was higher than that in the Altay and Tacheng [14] and the Changji eye studies [13] but lower than that in the Lhasa [11] and Luxi eye studies [13].

The univariate logistic regression analysis demonstrated that age, sex, education level, and BMI were significantly associated with low vision and blindness (*P* ≤ 0.001, =0.021, ≤0.001, ≤0.001, respectively), whereas smoking and drinking did not statistically correlate with low vision and blindness (*P*=0.221, 0.307). The multiple logistic regression showed that age (OR 1.095, 95% CI 1.077 to 1.113) was a risk factor associated with low vision and blindness, while education with ≤ primary school (OR 0.674, 95% CI 0.483 to 0.939), junior middle school (OR 0.608, 95% CI 0.382 to 0.967), and BMI (OR 0.938, 95% CI 0.901 to 0.977) were protective factors for low vision and blindness (Table 4).

The main causes of low vision were cataract (42.6%), refractive error (19.6%), glaucoma (12.6%), AMD (3.9%), and pterygium (3.9%), while the primary causes of blindness were cataract (34%), glaucoma (34%), retinitis pigmentosa (10%), AMD (6%), and refractive error (6%) (Table 5). Suggestions were provided for subjects with VI to further treat any pathological conditions.

## 4. Discussion

This study provides new population-based data on the prevalence, risk factors, and major causes of VI in Uygur populations in China. Our results showed that, compared with other minority population-based surveys in China, the prevalence of low vision and blindness was higher than that in northern Xinjiang, lower than that in Luxi, and similar to that in Tibet. Age, education, and BMI were significantly related to low vision and blindness. Cataract was the leading cause of low vision and blindness.

Overall, the study showed that the prevalence of low vision and blindness increased with age, which was evidenced by the prevalence of 8.7% among people aged 50 to 59 years, 23.1% among people aged 60 to 69 years, 44.2% among people aged 70 to 79 years, and 76.3% among people aged over 80 years. These results were in line with most other population-based surveys, which revealed that the prevalence estimates were higher among older populations [6, 7, 10, 12, 16–18].

The prevalence of low vision and blindness across China was 11.96% according to the China Nine-Province Survey, with the lowest rate in Beijing (6.71%) and the highest rate in Yunnan (20.05%) [7]. In the present study, the prevalence was 19.11%, which was remarkably higher than that in the former study. Inequalities in socioeconomic development, eye care program accessibility, and blindness prevention awareness among these provinces may be responsible for the differences. Compared with the few published surveys of Chinese minority groups, this kind of VI was increased. For example, the rate of low vision and blindness was 6.5% in Altay and Tacheng [14] and 9.35% in Changji [13]. One possible explanation is that southern Xinjiang has a less developed economy than northern Xinjiang. Therefore, professional health care resources and facilities are not easily accessible in this region. The other speculated reason is that subjects with mixed ethnicity who were not purely Uygur were enrolled in those studies. The similar prevalence to that in Tibet (18.84%) is probably attributable to the nearly parallel economic levels between the regions. The prevalence of low vision and blindness (19.11%) was slightly lower than that in Yunnan Province (21.24%) in the China Nine-Province Survey [13]. This was possibly associated with the latter study being conducted more than 10 years ago. Although two epidemiological surveys of Chinese Bai and Dai ethnicities in Yunnan Province were conducted [10, 12], they did not directly report the rate of moderate VI. However, the reported prevalence of worse than mild VI (32.95% and 31.2%, respectively) approached our value of 32.4%. Moreover, the prevalence of low vision and blindness was 3.53% and 2.75%, respectively, in Taiwan [19, 20] and 8.9% in Taizhou [21]. These lower rates largely occurred because the VI criteria were quantified by best-corrected visual acuity (BCVA) instead of PVA, and some participants aged 45–49 years were also enrolled. The worldwide prevalence also varied substantially. Therefore, it was difficult to accurately compare this population with those in other countries because of differences in economic development, study sites (rural or urban), examination dates, definitions of VI, and sample sizes. For example, the prevalence of low vision and blindness was 25% in the Wurno health zone in 2016 [22] and 40.4% in rural Myanmar in 2005 [23], while the prevalence of low vision in an urban population aged 60–80 years in Copenhagen city was only 1.91% from 1986 to 1988 [24]. The global prevalence of low vision and blindness decreased from 1990 to 2010 [25]. Nevertheless, the low-vision population may increase due to aging.

Published studies have demonstrated that a lack of education tends to be associated with low vision or blindness [6, 10]. In the current study, 95.3% of the subjects had an education level lower than high school. The multiple logistic regression showed that the educated participants had a lower risk of low vision and blindness than the uneducated participants, although statistical significance was not found in participants with a high school or higher education level (*P*=0.181). This finding was expected and consistent with many previous population-based studies [7, 10, 13, 26, 27]. Variations in the level of eye health care awareness among these people may account for the disparity. People with relatively high education levels may be more aware of eye health than those with relatively low education levels. Therefore, attention should be paid to those with low education levels during the development and implementation of blindness prevention strategies.

Interestingly, high BMI was a protective factor for low vision and blindness, as people with higher BMIs were less likely to suffer from low vision or blindness than those with lower BMIs. There are few studies on the effect of BMI on VI, and a consensus has not been reached. Cui et al. reported that VI was negatively associated with BMI based on PVA or BCVA in patients with type 2 diabetes [28]. However, Kahloun et al. showed that VI in diabetic patients was significantly associated with a BMI >25 [29]. In the current study, the average BMI (25.3 kg/m^2^) in subjects with normal vision was significantly higher than that in subjects with low vision and blindness. However, given that a BMI of 22.3–23.8 kg/m^2^ was still in a normal range, the reason underlying the significant association remained unclear, and the relationship between BMI and VI needs to be confirmed in the future.

Cataract is the predominant cause of low vision or blindness in China, including Taiwan [19], Taizhou [21], Bin County [6], and Yunnan [10], as well as in many other countries, including Kumejima Island in Japan [30], Meiktila in Myanmar [23], India [31], Singapore [32], and Nepal [33]. In the current study, cataract was the most common reason for low vision and blindness, accounting for 42.6% of the low vision and 34% of the blindness. This finding indicated that the government and hospitals should make more effort to increase the cataract surgery rate to reduce the prevalence of this easily treatable disease.

Refractive error accounted for 19.6% of the low-vision participants, which indicated a lack of eye care resources among the residents. Only 6% of the blindness was caused by refractive error, which was almost consistent with the participants' low education levels. This was similar to some other studies in which refractive error was not the primary cause of low vision or blindness [18, 30, 34].

In addition, 12.6% of the low-vision cases and 22% of the blindness cases were caused by glaucoma. This finding was higher than that in the elderly Bai ethnic group in Yunnan [10], in which glaucoma was responsible for 3.1% of the low vision and 5.3% of the blindness based on PVA, and was similar to the Shihpai Eye Study [19], which reported that 8.3% of subjects with a BCVA<20/60 was caused by glaucoma. The higher prevalence of glaucoma could be associated with the higher prevalence of exfoliation syndrome in the region, which increased the risk for glaucoma. In the current study, among the 29 glaucoma subjects with low vision, 11 (37.9%) had exfoliation glaucoma, whereas, among the 11 glaucoma subjects with blindness, 5 (45.5%) had exfoliation glaucoma. This was similar to the published studies which showed exfoliation syndrome as a significant risk factor for glaucoma; for example, glaucoma was about 8-fold more common in eyes with exfoliation syndrome compared to those without it [35].

The strengths of the current study included the use of a population-based survey, the satisfactory response rate, and the epidemiological analysis of VI in population of only Uygur nationality. Still, there were several limitations. First, BCVA and automatic refraction were not performed. However, WHO recommends using PVA to evaluate VI because BCVA overlooks many people with VI. Second, the sample size is not large enough. Nevertheless, data of the Uygur population have been limited thus far, and this study contributes information regarding the epidemiology of VI in this population in China.

## 5. Conclusions

In conclusion, our study shows that VI is an important public health issue among elderly Uygur individuals in southern Xinjiang in China, especially for those with low education levels. Cataract is the leading cause of low vision and blindness.

## Figures and Tables

**Table 1:** Demographic characteristics of the participants with different visual impairment levels.

Parameters	Total (n)	Normal vision	Mild visual impairment	Moderate visual impairment	Severe visual impairment	Blindness	*P*
Age (years)	1465	56.7 ± 8.5	60.9 ± 8.6	65.4 ± 9.8	70.1 ± 10.6	67.9 ± 11.7	≤0.001
Sex (%)							
Male	673	447	80	93	26	27	
Female	792	543	115	94	17	23	
							0.085
Education (n)							
Uneducated	323	177	49	64	15	18	
≤primary school	817	563	115	91	24	24	
Junior middle school	256	197	24	26	3	6	
High school or higher	69	53	7	6	1	2	
							≤0.001
BMI (kg/m^2^)	1465	25.3 ± 4.0	24.6 ± 3.6	23.8 ± 4.1	22.3 ± 3.6	22.9 ± 3.2	≤0.001
Smoking							
Yes	161	116	20	17	5	3	
No	1304	874	175	170	38	47	
							0.623
Drinking							
Yes	33	27	2	3	1	0	
No	1432	963	193	184	42	50	
							0.426

BMI: body mass index; kg/m^2^: kilogram/meter^2^.

**Table 2:** Age-specific and gender-specific prevalence of visual impairment.

Group	At risk (*n*)	Normal vision (%)	Mild visual impairment (%)	Moderate visual impairment (%)	Severe visual impairment (%)	Blindness (%)
Age (years)						
50–59	813	661 (81.3)	81 (10.0)	54 (6.6)	8 (1.0)	9 (1.1)
60–69	433	248 (57.3)	85 (19.6)	70 (16.2)	13 (3.0)	17 (3.9)
70–79	181	76 (42)	25 (13.8)	48 (26.5)	14 (7.7)	18 (9.9)
80+	38	5 (13.2)	4 (10.5)	15 (39.5)	8 (21.1)	6 (15.8)
Total	1465	990 (67.6)	195 (13.3)	187 (12.8)	43 (2.9)	50 (3.4)
Sex						
Male	673	447 (66.4)	80 (11.9)	93 (13.8)	26 (3.9)	27 (4.0)
Female	792	543 (68.6)	115 (14.5)	94 (11.9)	17 (2.1)	23 (2.9)
Total	1465	990 (67.6)	195 (13.3)	187 (12.8)	43 (2.9)	50 (3.4)

**Table 3:** Comparisons of the prevalence of VI or blindness with those in other minority population-based studies.

Location	Current study	Lhasa Eye Study	Xishuangbanna Eye Study	Dali Eye Study	Altay and Tacheng Eye Study	China Nine-Province Survey	China Nine-Province Survey
	Xinjiang	Tibet	Yunnan	Yunnan	Xinjiang	Luxi, Yunnan	Changji, Xinjiang
Year examined	2018	2010	2010	2010	2015	2006–2007	2006–2007
Setting	Rural	Urban and rural	Rural	Rural	Rural	Rural	Rural
Age range (years)	≥50	≥40	≥50	≥50	≥50	≥50	≥50
Ethnicity	Uygur	Zang	Dai	Bai	Han, Kazak, Uygur, and others	Han, Yi, Hui, Zhuang, Miao, Dai	32 ethnicities
Moderate VI	12.76%	10.41%	23.8%^*∗*^	24.82%^*∗*^	3.60%	11.96%	7.41%
Severe VI	2.94%		7.40%	8.13%	1.20%	3.88%	0.61%
Blindness	3.41%	8.43%			1.70%	5.40%	1.33%

∗Mild and moderate VI, <20/40 to >20/200; VI: visual impairment.

**Table 4:** Univariate and multiple logistic regression analysis of risk factors associated with low vision and blindness.

Parameters	Low vision and blindness			
	Univariate		Multiple	
	Or (95% CI)	*P*	Or (95% CI)	*P*
Age (years)	1.106 (1.089–1.123)	≤0.001	1.095 (1.077–1.113)	≤0.001
Sex	0.736 (0.567–0.956)	0.021	0.970 (0.739–1.274)	0.827
Education				
Uneducated	Reference	Reference	Reference	Reference
≤Primary school	0.478 (0.354–0.645)	≤0.001	0.674 (0.483–0.939)	0.020
Junior middle school	0.369 (0.240–0.566)	≤0.001	0.608 (0.382–0.967)	0.036
High school or higher	0.349 (0.167–0.732)	0.005	0.584 (0.265–1.284)	0.181
BMI (kg/m^2^)	0.882 (0.850–0.916)	≤0.001	0.938 (0.901–0.977)	0.002
Smoking	0.756 (0.483–1.183)	0.221	0.881 (0.523–1.485)	0.635
Drinking	0.578 (0.201–1.657)	0.307	1.263 (0.401–3.979)	0.690

OR: odds ratio; CI: confidence intervals; BMI: body mass index.

**Table 5:** Causes of low vision and blindness.

Major causes	Low vision ^#^(*n* = 230)	Blindness ^*∗*^ (*n* = 50)
Cataract	98 (42.6%)	17 (34%)
Glaucoma	29 (12.6%)	11 (22%)
Highly myopic retinopathy	5 (2.2%)	2 (4%)
Age-related macular degeneration	9 (3.9%)	3 (6%)
Refractive error	45 (19.6%)	3 (6%)
Optic atrophy	4 (1.7%)	2 (4%)
Retinitis pigmentosa	3 (1.3%)	5 (10%)
Amblyopia	5 (2.2%)	
Pterygium	9 (3.9%)	
Retinal venous occlusion	3 (1.3%)	
Others	10 (4.3%)	6 (12%)
Undetermined	10 (4.3%)	1 (2%)

^#^Low vision = PVA worse than 20/63 but better than 20/400 in the better eye. ^*∗*^Blindness = PVA worse than 20/400 in the better eye.

## Data Availability

The data used to support the findings of this study are available from the corresponding author upon request.
